# Insights into the origin of the high variability of multivalent-meiotic associations in holocentric chromosomes of *Tityus* (*Archaeotityus*) scorpions

**DOI:** 10.1371/journal.pone.0192070

**Published:** 2018-02-21

**Authors:** Viviane Fagundes Mattos, Leonardo Sousa Carvalho, Marcos André Carvalho, Marielle Cristina Schneider

**Affiliations:** 1 Universidade Estadual Paulista “Júlio de Mesquita Filho”, UNESP, Departamento de Biologia, Rio Claro, São Paulo, Brazil; 2 Universidade Federal do Piauí, UFPI, Campus Almícar Ferreira Sobral Floriano, Piauí, Brazil; 3 Universidade Federal de Mato Grosso, UFMT, Departamento de Biologia e Zoologia, Cuiabá, Mato Grosso, Brazil; 4 Universidade Federal de São Paulo, UNIFESP, Departamento de Ecologia e Biologia Evolutiva, Diadema, São Paulo, Brazil; University of Arkansas, UNITED STATES

## Abstract

Scorpions represent an intriguing group of animals characterized by a high incidence of heterozygous chromosomal rearrangements. In this work, we examined six species of *Tityus* (*Archaeotityus*) from Brazilian fauna with a particular focus on elucidating the rearrangements responsible for the intraspecific variability of diploid number and the presence of long chromosomal chains in meiosis. To access any interpopulation diversity, we also studied individuals from four species representing distinct localities. Most species demonstrated intraspecific polymorphism in diploid number (2n = 19 and 2n = 20 in *T*. *clathratus*, *T*. *mattogrossensis*, and *T*. *pusillus*, 2n = 16, 2n = 17 and 2n = 18 in *T*. *paraguayensis*, and 2n = 16 and 2n = 24 in *T*. *silvestris*) and multi-chromosomal associations during meiosis I, which differed even among individuals with the same chromosome number. In some species, the heterozygous rearrangements were not fixed, resulting such as in *Tityus clathatrus*, in 11 different chromosomal configurations recognized within a same population. Based on meiotic chromosome behaviour, we suggested that independent rearrangements (fusion/fission and reciprocal translocations), occurring in different combinations, originated the multi-chromosomal chains. To evaluate the effects of these chromosome chains on meiotic segregation, we applied the chi-square test in metaphase II cells. The non-significant occurrence of aneuploid nuclei indicated that non-disjunction was negligible in specimens bearing heterozygous rearrangements. Finally, based on our analysis of many chromosome characteristics, e.g., holocentricity, achiasmate meiosis, endopolyploidy, ability to segregate heterosynaptic or unsynapsed chromosomes, (TTAGG)n sequence located in terminal regions of rearranged chromosomes, we suggest that the maintenance of multi-chromosomal associations may be evolutionarily advantageous for these species.

## Introduction

Karyotype comparisons between many species of plants and animals have revealed large-scale chromosomal differences [1–7]. Additionally, new technologies, such as genome scanning have shown that submicroscopic chromosomal variants are more common than previously thought [8–10]. These microscopic and submicroscopic chromosomal variants play important roles in species evolution, promoting changes in gene expression and influencing aspects of local adaptation [11–17]. Rearrangements that give rise to chromosome variants frequently reduce the fertility of hybrids due to problems related to chromosome segregation in meiosis and the generation of unbalanced gametes via duplication or deletion. However, unbalanced gametes are suppressed when chromosomally rearranged regions are protected from recombination [14, 17].

Karyotype changes originate from deletions, duplications, inversions or translocations occur in both holocentric and monocentric chromosomes [15, 18]. In monocentric chromosomes, rearrangements sometimes produce acentric or dicentric fragments, which are frequently lost during cell division. In contrast, in holocentric, all chromosome segments arising from rearrangements might segregate normally to the poles due to the attachment of microtubules along the entire chromosome length [19–21].

Chromosomal heterozygosity originating from translocation, fusion or fission leads to the formation of trivalent, quadrivalent, or even multivalent chromosomal associations during meiosis I. Multivalent associations of more than four chromosomes are rare or underestimated, although they have been observed in certain plants [22–24], invertebrates [25–30] and vertebrates [31–36]. These multi-chromosomal associations form via rearrangements involving both autosomes and sex chromosomes or autosomes alone. Generally, multivalent associations encompassing sex chromosomes appear as a linear chain, and those formed only by autosomes have a ring configuration [28, 33, 36, 37]. In some cases, numbers of chromosomes involved in multivalent associations vary within a species, as observed in the cockroach *Periplaneta americana*, which presents 2n = 33 chromosomes organized in rings, representing multivalent associations of four, six or eight chromosomes [25, 38]. The huntsman spider, *Delena cancerides*, has 2n = 22–43 chromosomes in linear chains comprised of three to 19 chromosomes [28]. Intraindividual variations have been rarely documented, but examples include one population of the monocot plant *Allium roylei* with 2n = 16 chromosomes and cells containing one trivalent as well as other cells containing complex multivalent associations comprising all chromosomes [22].

Scorpions represent an intriguing group of animals characterized by a high incidence of heterozygous chromosomal rearrangements. In addition, the family Buthidae combines three unusual cytogenetic features: holocentric chromosomes, multivalent-meiotic associations and achiasmate meiosis in males [27, 29, 39–42]. Multivalent associations in meiosis have been observed in 50% of the species included in eight different buthid genera [27, 29, 39–48]. The numbers of chromosomes involved in these chains vary within a species, e.g., *Isometrus maculatus* (2n = 12) with multivalent of IV, VI and VIII chromosomes [43–45]; *Lychas marmoreus* (2n = 12–15) with seven bivalents (II) and chains consisting of the IV, VI, VIII, and X chromosomes [27, 49]; *Jaguajir agamemnon* (2n = 28) with the 14II and chains of XXVIII chromosomes; and *Jaguajir rochae* (2n = 28) with VIII, X and IV+VIII chromosomes in the chains [29]. However, the most extreme variability in chromosomal configurations has been observed in the genus *Tityus*. In *T*. *bahiensis* (2n = 5–19), not only are there differences in specimens with the same diploid number (2n = 9: III, IV, VII; 2n = 10: III, IV, VI, VII, X) [39, 50–54], but variations among cells from the same individual have also been reported, i.e., one specimen with 2n = 6 presented cells with three well-paired elements resembling bivalents and others with one multivalent association consisting of six chromosomes [39]. A single *T*. *paraguayensis* individual (2n = 16) exhibited cells with 8II as well as polyploid cells containing chromosomal chains composed of a variable number of elements (5II+3IV+VIII) [29].

In buthid scorpions, heterozygous reciprocal translocations or fission/fusion rearrangements are hypothesized to be responsible for the origin of multi-chromosomal associations during meiosis I [27, 29, 39–42, 48]. The mechanisms responsible for correct chromosome pairing and segregation of long chromosomal chains remain poorly characterized. However, permissive meiosis, which facilitates the correct alignment and alternate segregation of chromosomes involved in complex chains, is necessary to maintain fertility and produce balanced gametes [28, 33]. Moreover, only a small number of species have routinely demonstrated multi-chromosomal association in meiosis; thus, there appears to be a genetic ability to balanced chromosomal chain segregation [28, 33].

**Fig 1:**
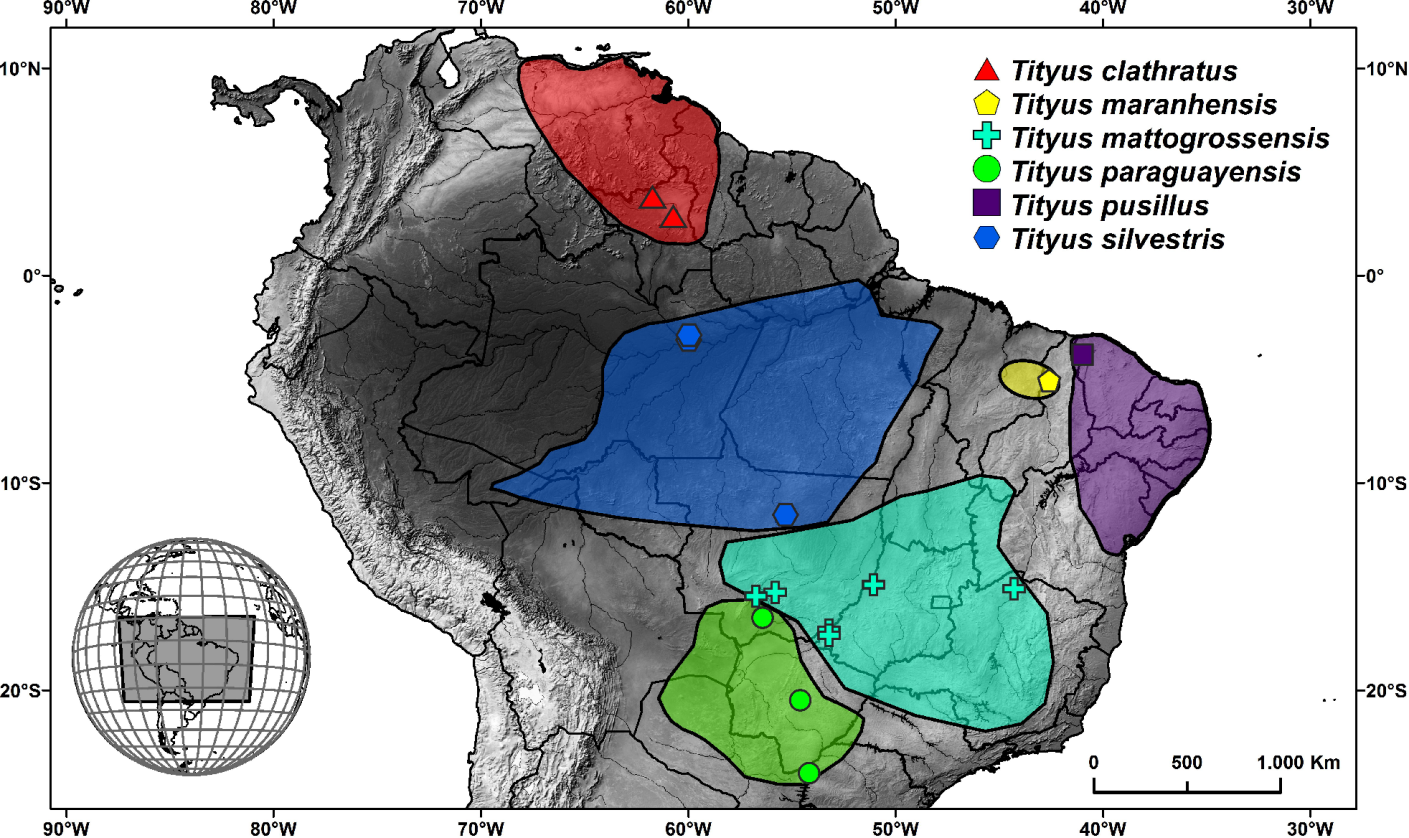
Distribution and collection localities in Brazil of the scorpion species analyzed in this work. Species distribution was based on Lourenço [55–60] and Lourenço et al. [61].

In this work, we examined six species of *Tityus* (*Archaeotityus*) from Brazilian fauna using standard and molecular cytogenetic techniques with a particular focus on elucidating the rearrangements responsible for the intraspecific variability of diploid number and the presence of long chromosomal chains in meiosis. To access any interpopulation diversity, we also studied individuals from four species representing distinct localities, as shown in Fig 1.

## Material and methods

We investigated a sample of 137 scorpions, all of which belonged to the subgenera *Tityus* (*Archaeotityus*), specifically *T*. *clathratus*, *T*. *maranhensis*, *T*. *mattogrossensis*, *T*. *paraguayensis*, *T*. *pusillus* and *T*. *silvestris*. Data describing the numbers of analyzed individuals for each species and the collection localities are listed in Table 1. Collecting permits from all localities were granted by the Instituto Brasileiro de Meio Ambiente e Recursos Naturais Renováveis (IBAMA) and the Instituto Chico Mendes de Conservação da Biodiversidade (ICMBIO) (23561–1; 25471–1; 40014–2; 41383–2; 48224–1). Scorpion species within the subgenus *Archaeotityus* present a rather similar morphology, with variegated pigmentation and a very rhomboidal subaculear tooth [59]. However, the group has not undergone a robust taxonomic revision, and the interspecific morphological differences are narrow, thus hampering species recognition. The scorpions were identified using relevant taxonomic literature [55–61] by the authors and confirmed by Jairo Andrés Moreno-Gonzales (Universidade de São Paulo, state of São Paulo, Brazil). Vouchers were deposited in three Brazilian collections: Coleção de História Natural of the Universidade Federal do Piauí (CNHUFPI–curator E.F.B. Lima), Floriano, state of Piauí; Coleção Zoológica of the Universidade Federal de Mato Grosso (UFMT—curator A. Chagas-Jr), Cuiabá, state of Mato Grosso; and Coleções Taxonômicas of the Universidade Federal de Minas Gerais (UFMG–curator A.J. Santos), Belo Horizonte, state of Minas Gerais.

**Table 1:** *Tityus* scorpions analyzed in this work, including numbers of specimens and the collection localities in Brazilian states.

Species	Number of individuals	Collection localities
*Tityus clathratus* C.L. Koch, 1844	14♂/1♀	Amajari (3°47’N, 61°43’W), RR
	1♂	Boa Vista (2°52’N, 60°43’W), RR
*Tityus maranhensis* Lourenço, de Jesus Junior and Lima de Oliveira, 2006	5♂	Floresta Nacional dos Palmares (5°03’S, 42°35’W), Altos, PI
*Tityus mattogrossensis* Borelli, 1901	9♂	Alto Araguaia, A (17°17’S, 53°13’W), MT
	12♂	Alto Araguaia, B (17°08’S, 53°12’W), MT
	2♂	Aruanã (14°55’S, 51°04’W), GO
	11♂	Cuiabá (15°29’S, 56°43’W), MT
	2♂	Parque Nacional Cavernas do Peruaçu (15°07’S, 44°16’W), Januária, MG
	5♂/3♀	Parque Nacional Chapada dos Guimarães (15°17’S, 55°48’W), Chapada dos Guimarães, MT
*Tityus paraguayensis* Kraepelin, 1895	1♂	Base de Estudos do Pantanal (16°30’S, 56°25’W), Poconé, MT
	1♂	Ilha Rodrigues Saraiva (24°01’S, 54°10’W) Guaíra, PR
	1♂	Ilha São Francisco (24°01’S, 54°10’W), Guaíra, PR
	26♂	Universidade Federal de Mato Grosso do Sul (20°29’S, 54°36’W), Campo Grande, MS
*Tityus pusillus* Pocock, 1893	23♂/1♀	Serra da Ibiapaba (3°49’S, 40°59’W), Ubajara, CE
*Tityus silvestris*Pocock, 1897	9♂/3♀	Fazenda Continental (11°34’S, 55°17’W), Claudia, MT
	2♂	Reserva Florestal Adolpho Ducke (2°55’S, 59°58’O), Manaus, AM
	5♂	Universidade Federal do Amazonas (3°06’S, 59°58’W), Manaus, AM
**Total**	129♂/8♀	

AM = Amazonas, CE = Ceará, GO = Goiás, MG = Minas Gerais, MS = Mato Grosso do Sul, MT = Mato Grosso, PI = Piauí, PR = Paraná, RR = Roraima.

Chromosomal preparations to study mitotic and meiotic chromosomes were obtained according to the technique described by Schneider et al. [39] and standard-stained with 3% Giemsa solution. Active nucleolar organizer regions (Ag- NORs) were detected by silver impregnation [62]. To locate major ribosomal cistrons and telomeric sites, the preparations were subjected to fluorescent in situ hybridization (FISH) following the procedure of Pinkel et al. [63] with modifications as described. The 28S rDNA probes were obtained by PCR using the genomic DNA of *T*. *silvestris* and the primers 28S-F 5’ GACCCGTCTTGAAACACGGA and 28S-R 5’ TCGGAAGGAACCAGCTACTA as described by Nunn et al. [64]. Telomeric probes were generated by PCR without DNA template using only the primers Tel-F 5’ TAGGTTAGGTTAGGTTAGG and Tel-R 5’ AACCTAACCTAACCTAACC. In both cases, probes were labeled with biotin-16-dUTP (1mM) by PCR and detected with anti-biotin conjugated to Alexa Fluor 488 (200μg/mL). For FISH, slides were incubated in 1% pepsin solution and RNAse/2xSSC (40 μg/mL) for 1h at 37°C. The chromosomes were fixed in 1% formaldehyde (1xPBS/50mM MgCl_2_) for 10 min, washed in 1xPBS for 5 min and dehydrated using an ethanol series (70%, 90% and 100%) for 5 min per step. Chromosomal DNA was denatured in 70% formamide/2xSSC for 3 min and 30 s at 70°C and dehydrated in an ice-cold ethanol series. The hybridization mixture (50% deionized formamide, 20% dextran sulfate, 10% 20xSSC, 20% DNA probe) was denatured in a thermocycler for 10 min at 95°C and applied to the slides, which were maintained in a humid chamber overnight at 37°C. Post-hybridization washes were performed twice in 15% formamide/2xSSC for 5 min each at 42°C and in 0.5% Tween for 5 min at room temperature. The chromosome spreads were counterstained with DAPI/Vectashield. The images of the mitotic and meiotic cells were captured in a Zeiss Imager A2 microscope coupled to a digital camera using Axio Vision software.

For the five cytogenetically analyzed species, *T*. *clathratus*, *T*. *mattogrossensis*, *T*. *paraguayensis*, *T*. *pusillus* and *T*. *silvestris*, all cells on each slide were photographed. The number of cells per individual and the chromosome configuration observed in postpachytene nuclei are described in Tables 2 and 3. Cells with a variable number of chromosomes and those in which chromosome configurations were not determined due to the complexity of the chromosome chain were categorized as “variable number” (VN). Diploid set length (DSL) was determined for the following: two males of *T*. *clathratus*, CHNUFPI 1853 (18 cells) and CHNUFPI 1863 (24 cells), which showed differences in the number of bivalents but equal numbers of chromosomes involved in multivalent associations; and three males of *T*. *mattogrossensis*, UFMT 1362 (27 cells), UFMT 1357 (14 cells) and UFMT 1375 (25 cells), which exhibited chains of four chromosomes. To establish DSL, postpachytene cells with a similar degree of chromosome condensation were measured using ImageJ software (Image Processing and Analysis in Java) developed at the Research Services Branch of the US National Institute of Mental Health. In *T*. *clathratus*, the values obtained for DSL were compared based on the coefficient of variation (CV). Due to the heterogeneous degree of chromosome condensation among the cells, CV values up to 20% were considered statistically significant [65]. In *T*. *mattogrossensis*, DSL values were compared by performing principal component analysis (PCA) using R (R Core Team, 2017). For specimens of *T*. *clathratus*, *T*. *mattogrossensis*, *T*. *paraguayensis* and *T*. *pusillus* with odd diploid numbers, the data obtained for metaphase II cells were statistically compared using the chi-square test (df = 1) (Table 4).

**Table 2:** Percentages and number of cells (parentheses) in the chromosome configurations in male postpachytene cells of *Tityus clathratus*.

Populations	Chromosome configuration in postpachytene cells													
*Tityus clathratus*	10II	1II+XIII	2II+XI	3II+X	3II+XI	3II+XIII	4II+IX	4II+X	4II+XI	5II+IX	5II+X	5II+XI	VN	TOTAL
*Amajari*– **2n = 19**														
CHNUFPI 1873	-	19.4 (7)	-	-	-	30.6 (11)	-	-	-	-	-	-	50.0 (18)	36
CHNUFPI 1863	-	-	23.7 (9)	-	15.8 (6)	-	-	-	18.4 (7)	-	-	-	42.1 (16)	38
UFMT 1350	-	-	-	-	16.7 (3)	-	-	22.2 (4)	22.2 (4)	-	-	-	38.9 (7)	18
CHNUFPI 1875, 1927	-	-	-	-	-	-	-	-	35.3 (36)	-	-	-	64.7 (66)	102
CHNUFPI 1858	-	-	-	-	-	-	-	13.5 (8)	15.3 (9)	-	-	-	71.2 (37)	59
CHNUFPI 1852	-	-	-	-	-	-	-	-	-	10.5 (9)	18.6 (16)	9.3 (8)	61.6 (53)	86
*Amajari*– **2n = 20**														
CHNUFPI 1853	-	-	-	16.9 (12)	-	-	-	11.3 (8)	-	-	21.1 (15)	-	50.7 (36)	71
*Amajari*– **2n =?**														
CHNUFPI 1850	-	-	-	-	-	-	8.8 (3)	17.6 (6)	-	-	-	-	73.6 (25)	34
*Boa Vista*– **2n = 20**							-	-	-	-	-	-		
CHNUFPI 1860	69.6 (16)			-	-	-	-	-	-	-	-	-	30.4 (7)	23

VN = variable number. II = bivalent. The Roman numeral indicates the number of chromosomes in the multivalent associations.

**Table 3:** Percentages and number of cells (parentheses) in the chromosome configurations in male postpachytene cells of *Tityus* species.

Species/ Populations	Chromosome configuration in postpachytene cells									
***Tityus mattogrossensis***	**6II+III+IV**		**8II+III**		**10II**		**8II+IV**		**VN**	**TOTAL**
*Alto Araguaia A*– **2n = 20**										
UFMT 1370, 1371, 1372, 1373, 1374	-		-		61.0 (83)		-		39.0 (53)	136
*Alto Araguaia B*– **2n = 20**										
UFMT 1376, 1377, 1378, 1379, 1380	-		-		69.6 (55)		-		30.4 (24)	79
UFMT 1375	-		-		-		69.5 (25)		30.5 (11)	36
*Aruanã* – **2n = 20**										
UFMT 1358, 1359	-		-		88.2 (30)		-		11.8 (4)	34
*Chapada dos Guimarães*– **2n = 20**										
UFMT 1360, 1361, 1363, 1364	-		-		72.0 (95)		-		28.0 (37)	132
UFMT 1362	-		-		-		92.6 (25)		7.4 (2)	27
*Januária*– **2n = 20**										
UFMT 1356	-		-		63.1 (60)		-		36.9 (35)	95
UFMT 1357	-		-		-		46.7 (21)		53.3 (24)	45
*Cuiabá* – **2n = 19**										
UFMT 1368	51.9 (14)		-		-		-		48.1 (13)	27
UFMT 1369	-		45.0 (9)		-		-		55.0 (11)	20
UFMT 1365, 1366, 1367	-		-		35.2 (19)		-		64.8 (35)	54
***Tityus paraguayensis***	**8II**		**9II**		**5II+VII**		**5II+VIII**		**VN**	**TOTAL**
*Poconé* – **2n = 16**										
UFMT 1351	46.7 (14)		-		-		-		53.3 (16)	30
*Guaíra*– **2n = 16**										
CHNUFPI1841	63.2 (12)		-		-		-		36.8 (7)	19
*Campo Grande*– **2n = 17**										
UFMT 1382, 1383, 1384, 1385, 1386	-		-		30.2 (49)		-		69.8 (113)	162
UFMT 1381	-		-		26.3 (26)		11.1 (11)		62.6 (62)	99
*Campo Grande*– **2n = 18**										
UFMT 1387, 1388, 1389, 1390, 1391,1392, 1393, 1394, 1395, 1396, 1397, 1398, 1399, 1400, 1401, 1402, 1403	-		80.0 (762)		-		-		20.0 (190)	952
***Tityus pusillus***	**10II**	**7II+IV**		**7II+V**	**7II+VI**	**8II+III**		**PO**	**VN**	**TOTAL**
*Ibiapaba*– **2n = 19**										
UFMG 15200	-	34.5 (31)		-	-	32.2 (29)		-	33.3 (30)	90
UFMT 1404	-	12.5 (5)		25.0 (10)	-	-		-	62.5 (25)	40
UFMT 1405	-	14.7 (11)		45.3 (34)	-	-		9.3 (7)	30.7 (23)	75
*Ibiapaba*– **2n = 20**										
UFMT 1406, 1407, 1408, 1409, 1410, 1411, 1412	85.6 (161)	-		-	-	-		-	14.4 (27)	188
UFMT 1413, 1414, 1415; UFMG 15198	-	-		-	51.3 (141)	-		-	48.7 (134)	275
*Ibiapaba*– **2n =?**										
CHNUFPI 1336; UFMG 15205, 15206, 15211	78.3 (18)	-		-	-	-		-	21.7 (5)	23
***Tityus silvestris***	**12II**				**2II+XII**				**VN**	**TOTAL**
*Cláudia*– **2n = 16**										
CHNUFPI 1804, 1806, 1816, 1818; UFMT 1353, 1354, 1355, 1416, 1417	-				36.5 (155)				63.5 (270)	425
*Manaus*, *Ducke*– **2n = 24**										
UFMT 1352; CHNUFPI 1835	28.6 (12)				-				71.4 (30)	42
*Manaus*, *UFAM*– **2n = 24**										
CHNUFPI 1795, 1796, 1797; 1474; 1476	47.5 (38)				-				52.5 (42)	80

VN = variable number. PO = polyploid cells. II = bivalent. The Roman numeral indicates the number of chromosomes in the multivalent associations.

**Table 4:** Statistical comparison between the numbers of metaphase II cells observed and expected in *Tityus* species.

Species	Diploid number and postpachytence configuration	Haploid number observed and expected in metaphase II cells		χχ2	PP value
*Tityus clathratus*		**n = 9**	**n = 10**		
	19 = 4II+XI	8 (10)	12 (10)	00.8	00.37
	19 = 4II+X; 4II+XI	11 (12)	13 (12)	00.16	00.68
*Tityus mattogrossensis*		**n = 9**	**n = 10**		
	19 = 10II	12 (12.5)	13 (12.5)	00.04	00.84
	19 = 8II+III	3 (5)	7 (5)	11.6	00.21
*Tityus paraguayensis*		**n = 8**	**n = 8**		
	17 = 5II+VII	5 (6.5)	8 (6.5)	00.7	00.40
	17 = 5II+VII; 5II+VIII	12 (10)	8 (10)	00.8	00.37
*Tityus pusillus*		**n = 9**	**n = 10**		
	19 = 7II+IV; 7II+V	6 (9)	12 (9)	22.0	00.16

The expected number is shown in parentheses. Degree of freedom = 1.

## Results

### Conventional Giemsa staining

The chromosomes of all species studied in this work (Table 1) were holocentric, exhibited synaptic and achiasmatic behavior during male meiosis I, and had multivalent associations in postpachytene cells. Intraspecific variabilities in diploid number and number of chromosomes involved in the chains were observed in the analyzed species (Tables 2 and 3). *Tityus maranhensis* showed chromosomal features similar to those previously reported by Mattos et al. [29]; thus, herein only the results obtained using molecular cytogenetic techniques are described.

**Fig 2:**
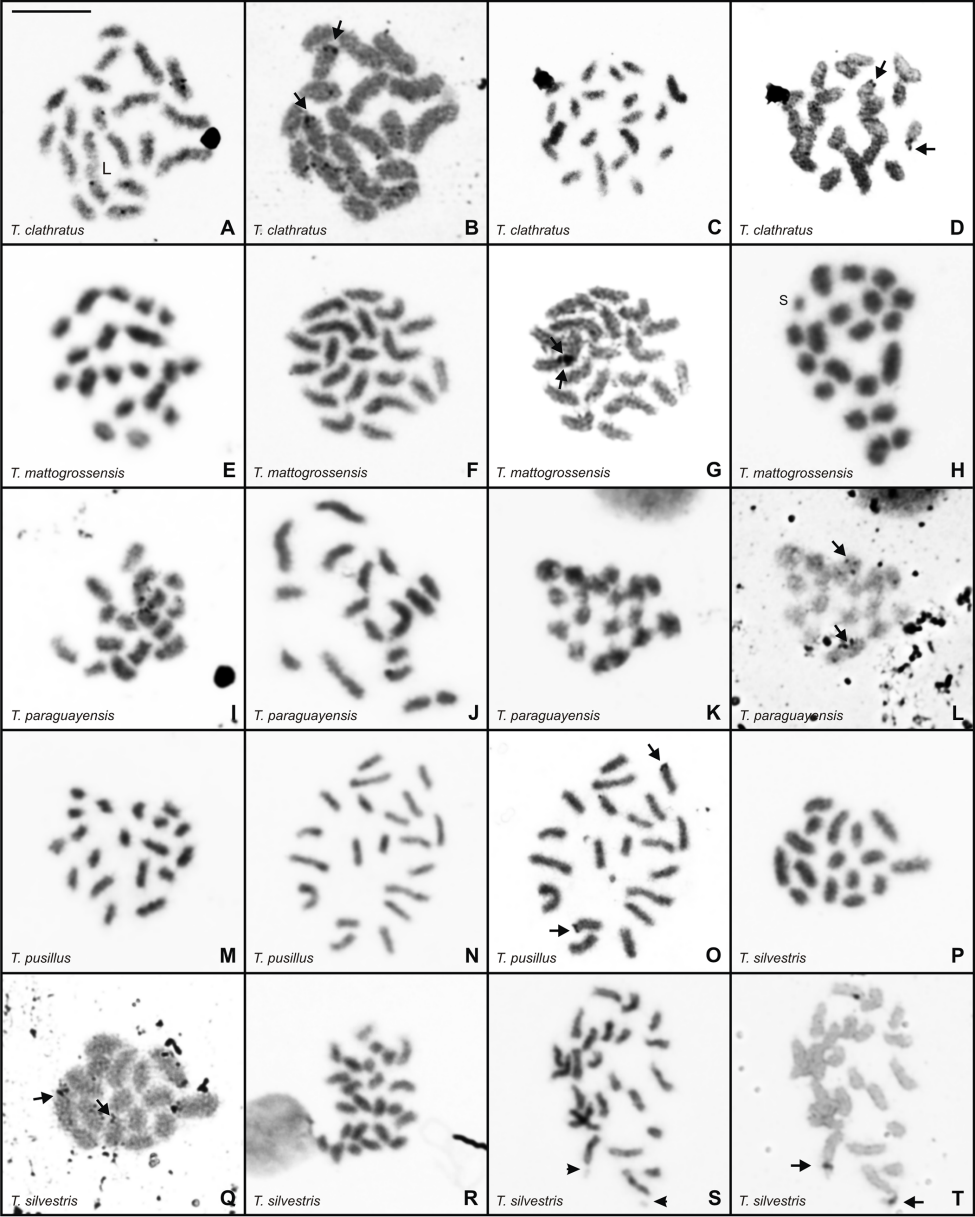
**Mitotic metaphase cells of *Tityus* species after Giemsa staining (**A, C, E, F, H, I, J, K, M, N, P, R, S) **and silver impregnation** (B, D, G, L, O, Q, T). (A-D) *Tityus clathratus*, 2n♂ = 19 (A-B) and 2n♂ = 20 (C-D). (E-H) *Tityus mattogrossensis*, 2n♂ = 19 (E) and 2n♂ = 20 (F-H). (i-l) *Tityus paraguayensis*, 2n♂ = 16 (I), 2n♂ = 17 (J) and 2n♂ = 18 (K-L). (M-O) *Tityus pusillus*, 2n♂ = 19 (M) and 2n♀ = 20 (N-O). (P-T) *Tityus silvestris*, 2n♂ = 16 (P-Q) and 2n♂ = 24 (R-T). In all species, the NORs (arrows) localized on the subterminal or terminal regions of two chromosomes. L = large chromosome size. S = small chromosome size. Arrowhead = constriction. Scale bar = 10μm.

Mitotic metaphase cells of five *Tityus* (*Archaeotityus*) species showed the following diploid numbers: 2n = 19 and 2n = 20 in *T*. *clathratus*, *T*. *mattogrossensis*, and *T*. *pusillus* (Fig 2A–2H, 2M–2O); 2n = 16, 2n = 17 and 2n = 18 in *T*. *paraguayensis* (Fig 2I–2L); and 2n = 16 and 2n = 24 in *T*. *silvestris* (Fig 2P–2T). Diploid number variability occurred within populations of *T*. *clathratus* from Amajari, *T*. *mattogrossensis* from Cuiabá, and *T*. *paraguayensis* from Campo Grande and between populations of *T*. *silvestris*. The chromosomes of all species gradually decreased in size (Fig 2). However, differences in chromosome size were verified in specimens of *T*. *clathratus* with 2n = 19, which presented one chromosome that was slightly larger than the other elements in the diploid set (Fig 2A); in two individuals of *T*. *mattogrossensis* from Cuiabá with 2n = 20, a single small-sized chromosome was identified (Fig 2H). Three individuals of *T*. *silvestris* (2n = 24) demonstrated terminal constrictions in two chromosomes (Fig 2S).

**Fig 3:**
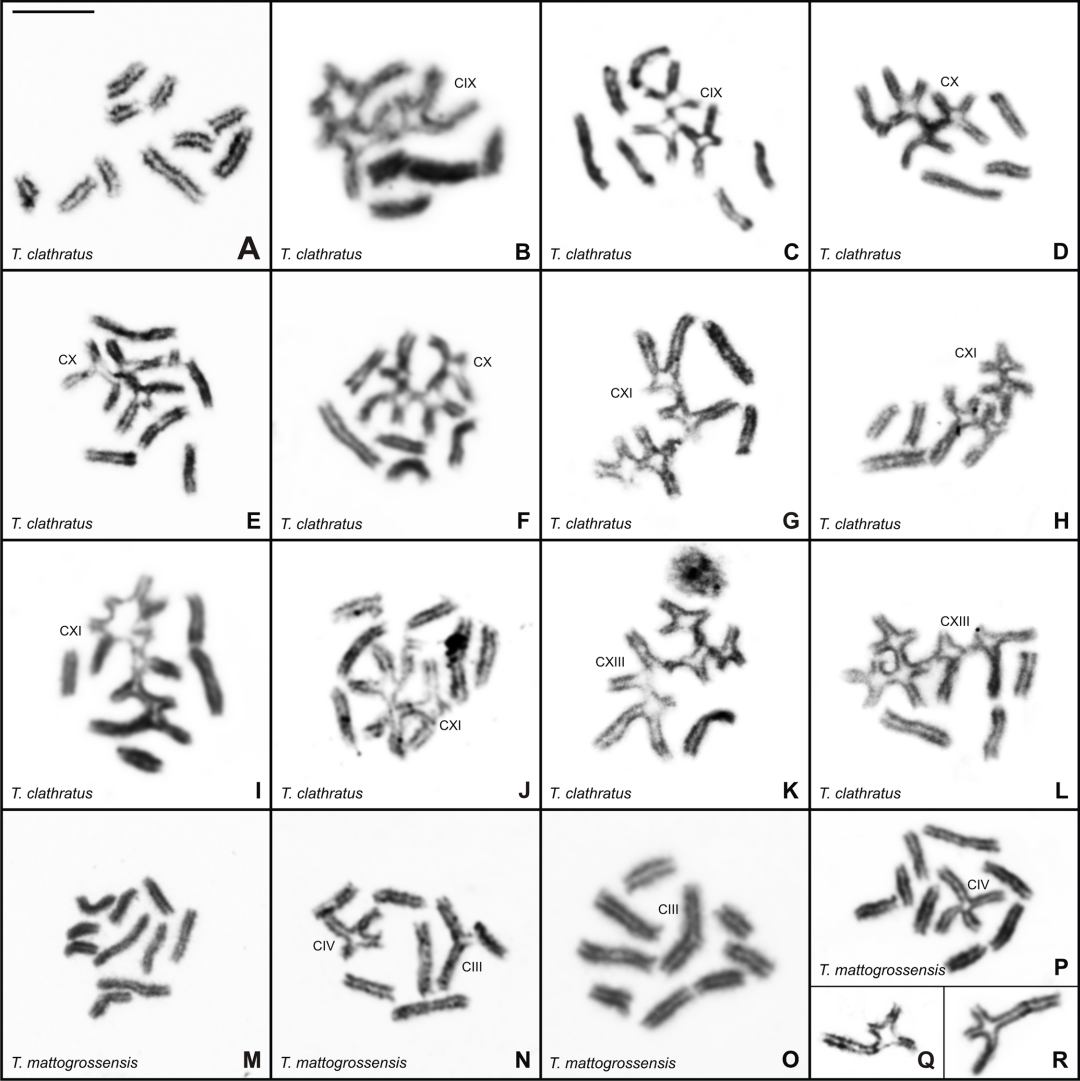
Postpachytene cells of *Tityus* stained with Giemsa. (A-L). *Tityus clathratus* with 10II (A), 4II+IX (B), 5II+IX (C), 3II+X (D), 4II+X (E), 5II+X (F), 2II+XI (G), 3II+XI (H), 4II+XI (I), 5II+XI (J), 1II+XIII (K), 3II+XIII (L). (M-R). *Tityus mattogrossensis* with 10II (M), 6II+III+IV (N), 8II+III (O), 8II+VI (P), and a quadrivalent association (Q, R). II = bivalent. The Roman numeral indicates the number of chromosomes in the chain. Scale bar = 10μm.

Unsynapsed interstitial and/or terminal chromosomal segments were observed in early pachytene cells of *T*. *clathratus* (2n = 19–20), *T*. *mattogrossensis* (2n = 19–20) and *T*. *paraguayensis* (2n = 17); these regions were generally continuous with completely paired segments. Total synapsis of the chromosomes was verified in late pachytene cells in which multivalent associations were also visualized (not showed). Some late pachytene cells of all species showed continuous and entirely synapsed chromosomes. The numbers and percentages of postpachytene cells with different chromosomal configurations were compiled in Tables 2 and 3. Among the five *Tityus* species, multi-chromosomal associations appeared closed in most postpachytene cells and open or completely disorganized in later substages of meiosis I (Fig 3).

**Fig 4:**
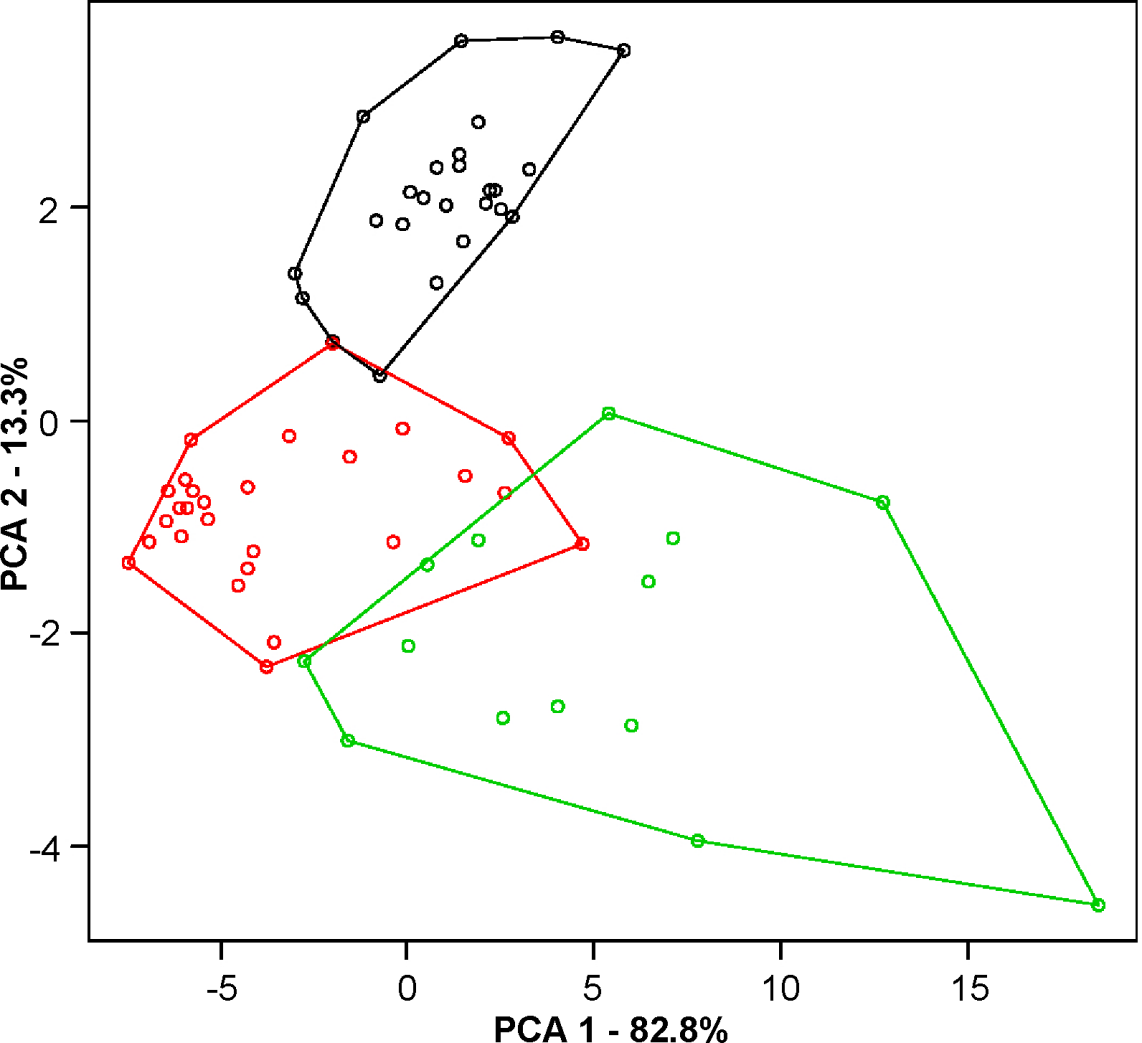
Analysis of length of the quadrivalent association of *Tityus mattogrossensis* from Alto Araguaia (black), Chapada dos Guimarães (red) and Januária (green). The chain of specimen from Januária differs from other two populations (scree plot 82.8%).

Postpachytene spermatocytes of *T*. *clathratus* from Boa Vista showed 10 bivalents with chromosomes disposed side by side (Fig 3A). In the sample from Amajari, 11 different chromosomal configurations were recognized in meiosis I (Table 2), including a variable number of bivalents and chains with nine (IX), 10 (X), 11 (XI) or 13 (XIII) chromosomes (Fig 3B–3L). The postpachytene configurations most frequently observed were 3II+XI and 4II+XI (Fig 3H and 3I) in specimens with 2n = 19, and 5II+X (Fig 4E) in males with 2n = 20. DSL was determined for two specimens of *T*. *clathratus* (CHNUFPI 1853; CHNUFPI 1863) that presented the same numbers of chromosomes in the chains but variable numbers of bivalents (see Table 2). The average DSL and the CV were as follows: specimen CHNUFPI 1853–147μm (3II+X), 163μm (4II+X), 175μm (5II+X), CV = 10.80; and specimen CHNUFPI 1863–139μm (2II+XI), 137μm (3II+XI), 179μm (4II+XI), CV = 15.86.Inlate postpachytene nuclei, the chains were organized in a zigzag manner, with homologous terminal chromosomes regions disposed in opposite sides, indicating correct orientation of the chromosomes for segregation in anaphase I.

The postpachytene cells of *T*. *mattogrossensis* with 2n = 19 presented three configurations: 10II, 6II+III+IV and 8II+III (Fig 3M–3O). Specimens with 2n = 20 exhibited 10II and 8II+IV (Fig 3P). Additionally, among the specimens with 8II+IV, the following differences regarding the sizes of chromosomes involved in the quadrivalent association were observed: 1) chain with chromosomes of similar size in males from Alto Araguaia (Fig 3P); 2) chain with two large and two medium-sized chromosomes in the specimen from Chapada dos Guimarães (Fig 3Q); and 3) chain with two large, one medium and one small-sized chromosomes in the male from Januária (Fig 3R). To verify whether differences in the sizes of these chromosomes were significant, DSL values were determined. The comparison of total DSL values was not significant, indicating no differences among individuals in terms of total chromosome length (bivalents and chromosomes in the chain). However, when we compared only the length of quadrivalents, the population from Januária differed from other two (Fig 4), revealing that chromosomes of distinct size were rearranged (scree plot on the x-axis 82.8%).

**Fig 5:**
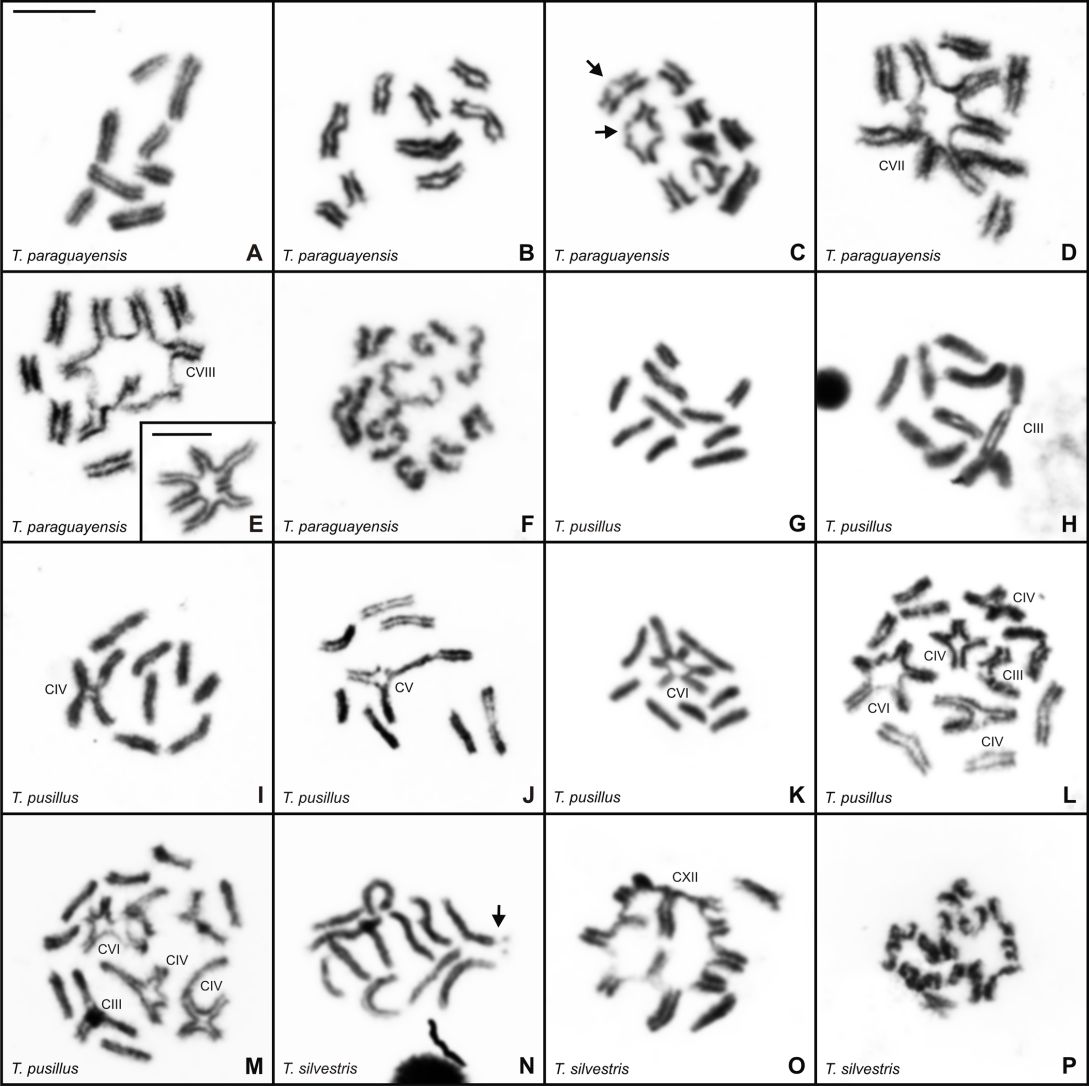
Meiotic cells of *Tityus* stained with Giemsa. (A-E, G-O) Postpachytene. (F, P) Anaphase I. (A-F) *Tityus paraguayensis* with 8II (A), 9II (B-C), 5II+VII (D), 5II+VIII (E). The detail in (E) highlights the chain of eight chromosomes. (G-M) *Tityus pusillus* with 10II (G), 8II+III (H), 7II+IV (I), 7II+V (J), 7II+VI (K), variable numbers of bivalents and chromosome chains, generally consisting of four chromosomes (L-M). (N-P) *Tityus silvestris* with 12II (N), 2II+XII (O), and the zigzag disposition of the chromosome chain (P). Arrow = gap or subterminal constriction. II = bivalent. The Roman numeral indicates the number of chromosomes in the chain. Scale bar = 10μm.

In *T*. *paraguayensis* with 2n = 16 and 2n = 18, postpachytene nuclei exhibited eight and nine bivalents, respectively (Fig 5A and 5B). In some cells, the bivalents revealed conspicuous regions with a low degree of condensation and the presence of gaps (Fig 5C). In specimens with 2n = 17, the configuration most commonly observed was 5II+VII (Fig 5D and 5E). In anaphase I, the total disassembly of the bivalents and multi-chromosomal associations was verified (Fig 5F).

*Tityus pusillus* specimens with 2n = 20 exhibited 10II with chromosomes disposed side by side and 7II+VI (Fig 5G and 5K). Numbers of bivalents and chromosome chains in males with 2n = 19 were categorized as 8II+III, 7II+IV and 7II+V (Fig 5H–5J). Additionally, one specimen (CHNUFPI 253) presented polyploidy in 9.33% of its postpachytene cells due to a larger number of bivalents and multi-chromosomal associations (Fig 5L and 5M).

Postpachytene nuclei of *T*. *silvestris* with 2n = 16 showed 2II+XII (Fig 5O). In late postpachytene cells, the zigzag disposition of chain chromosomes was verified (Fig 5P). All specimens with 2n = 24 presented 12II (Fig 5N). In some cells, a subterminal constriction was visible in one bivalent.

**Fig 6:**
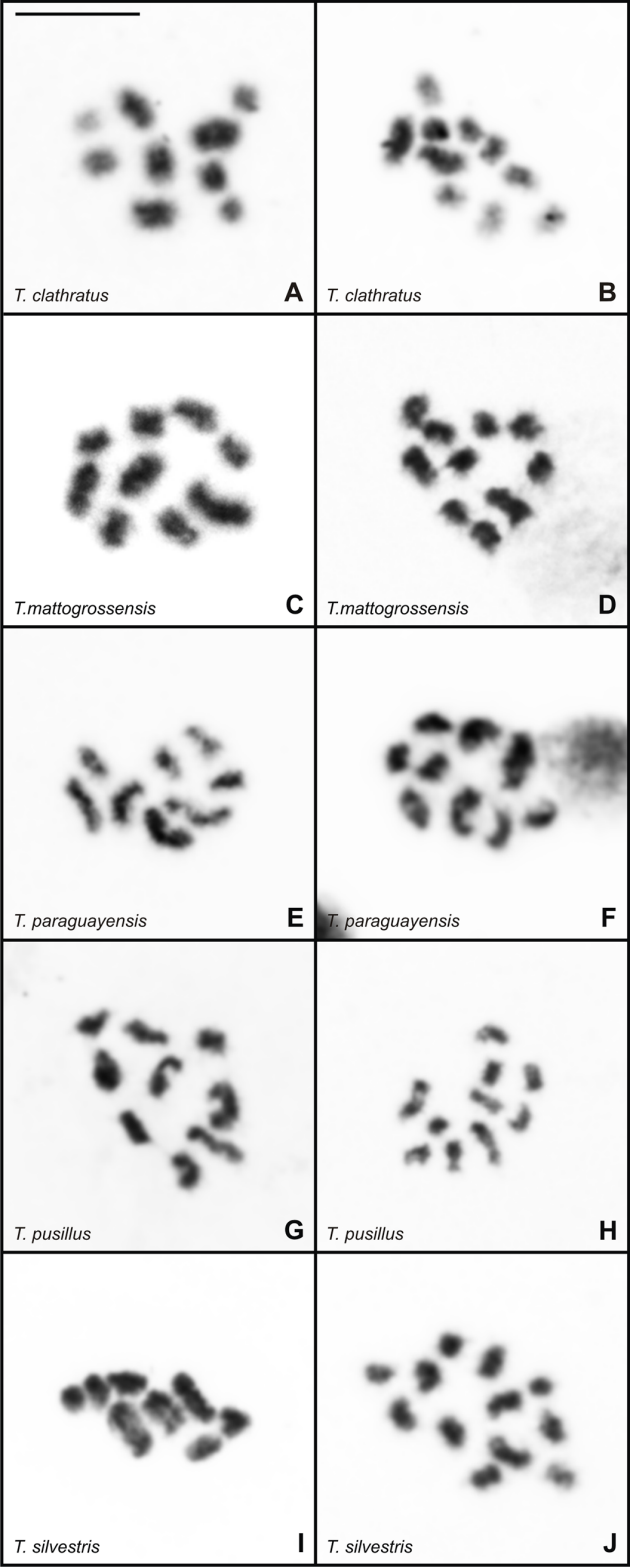
Metaphase II cells of *Tityus* stained with Giemsa. (A-B) *Tityus clathratus* with n = 9 (A) and n = 10 (B). (C-D) *Tityus mattogrossensis* with n = 9 (C) and n = 10 (D). (E-F) *Tityus paraguayensis* with n = 8 (E) and n = 9 (F). (G-H) *Tityus pusillus* with n = 9 (G) and n = 10 (H). (I-J) *Tityus silvestris* with n = 8 (I) and n = 12 (J). Scale bar = 10μm.

Among the five species, more than 70% of metaphase II nuclei exhibited the expected haploid number (Fig 6), i.e., n = 8 in *T*. *paraguayensis* (2n = 16) and *T*. *silvestris* (2n = 16); n = 8 and n = 9 in *T*. *paraguayensis* (2n = 17); n = 9 in *T*. *paraguayensis* (2n = 18); n = 9 and n = 10 in *T*. *clathratus* (2n = 19) and *T*. *pusillus* (2n = 19); n = 10 in *T*. *clathratus* (2n = 20) and *T*. *pusillus* (2n = 20); and n = 12 in *T*. *silvestris* (2n = 24). To evaluate the differences between the observed and expected haploid numbers, metaphase II cells of specimens with odd diploid numbers were subjected to the chi-square test. The results, shown in Table 4, indicate no statistically significant divergence.

### Ag-NORs and in situ hybridization

Mitotic metaphase cells of *T*. *clathratus*, *T*. *mattogrossensis*, *T*. *paraguayensis*, *T*. *pusillus* and *T*. *silvestris* revealed Ag-NORs localized on the subterminal or terminal regions of two chromosomes (Fig 2B, 2D, 2G, 2L, 2O, 2Q and 2T). In *T*. *clathratus*, *T*. *mattogrossensis* and *T*. *paraguayensis*, the NORs occurred on small-sized chromosomes compared with on medium-sized chromosomes in *T*. *pusillus* and *T*. *silvestris*. Additionally, association of the NORs with prominent terminal constriction was visible in *T*. *silvestris* (Fig 2T).

**Fig 7:**
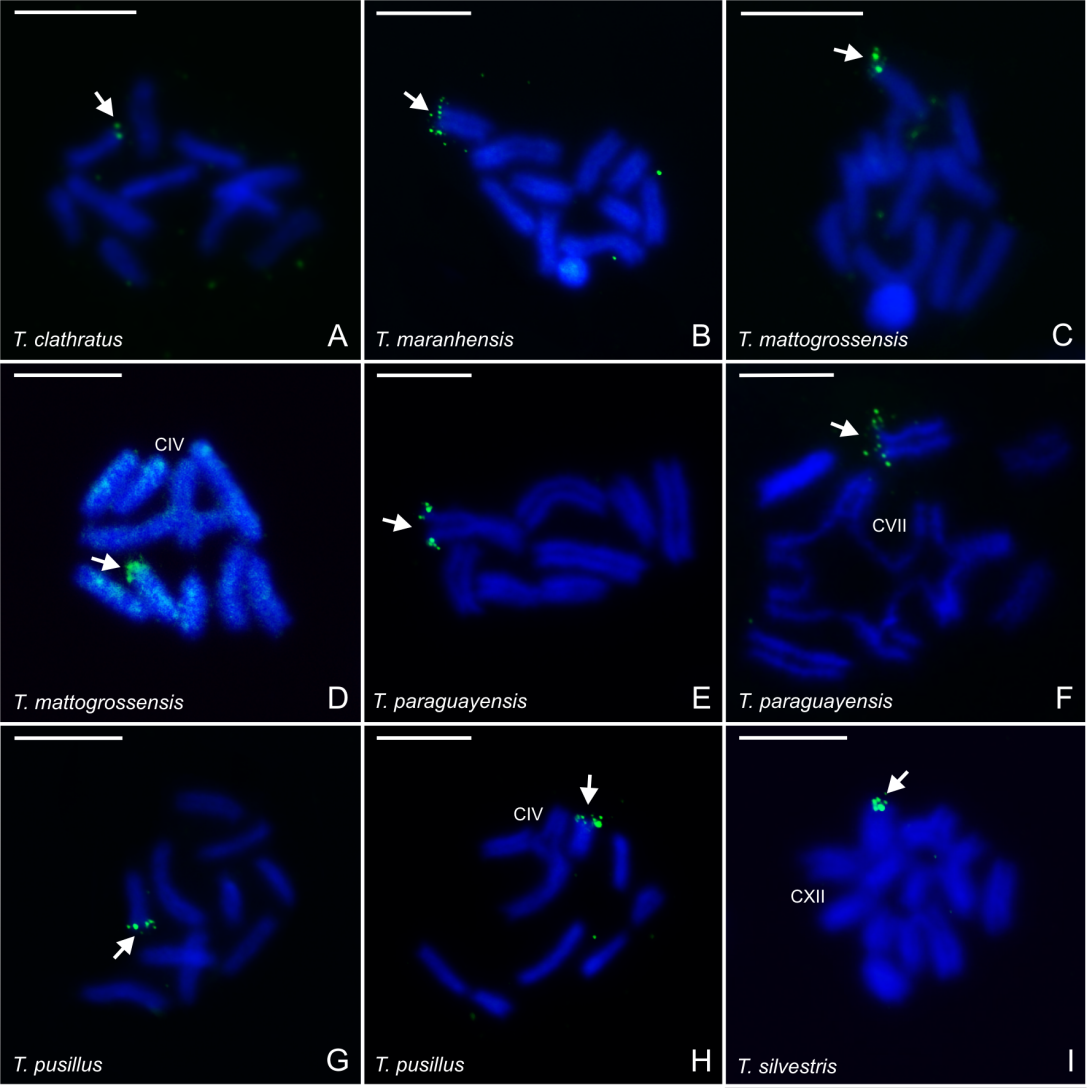
Localization of the 28S rDNA cistrons (green signal = arrow) in postpachytene chromosomes of *Tityus* species. (A) *Tityus clathratus* with 10II. (B) *Tityus maranhensis* 10II. (C-D) *Tityus mattogrossensis* with 10II (C) and 8II+IV (D). (E-F) *Tityus paraguayensis* with 8II (E) and 5II+VII (F). (G-H) *Tityus pusillus* with 10II (G) and 7II+IV (H). (I) *Tityus silvestris* with 2II+XII. II = bivalent. The Roman numeral indicates the number of chromosomes in the chain. Scale bar = 10μm.

The number of Ag-NORs was confirmed by FISH using a 28S rDNA probe (Fig 7). However, the analysis of postpachytene cells revealed that the ribosomal cistrons were not localized in well-paired meiotic elements resembling bivalents in all species. *Tityus clathratus* (10II) showed ribosomal cistrons in the terminal region of one bivalent (Fig 7A). Specimens of *T*. *maranhensis* (2n = 20) presented intraspecific variation in the number of bivalents (9II and 10II), but in both cases, only one bivalent was the carrier of the ribosomal cistrons (Fig 7B). The same pattern of 28S rDNA localization was observed in postpachytene nuclei of *T*. *mattogrossensis* with 10II and 8II+IV (Fig 7C and 7D), *T*. *paraguayensis* with 8II, 9II and 5II+VII (Fig 7E and 7F), and *T*. *pusillus* with 10II and 7II+IV (Fig 7G and 7H). However, *T*. *silvestris* with 2II+XII revealed ribosomal genes localized in two chromosomes involved in the multivalent association (Fig 7I). Mitotic metaphase cells demonstrated ribosomal sites in the terminal regions of two chromosomes. Frequently, there were differences in the sizes of ribosomal cistrons between homologues. In *T*. *silvestris* (2n = 24), the 28S rDNA sites were coincident with the constriction present in one chromosome pair. Metaphase II cells exhibited ribosomal sites localized in only one chromosome, indicating correct and balanced chromosomal segregation.

**Fig 8:**
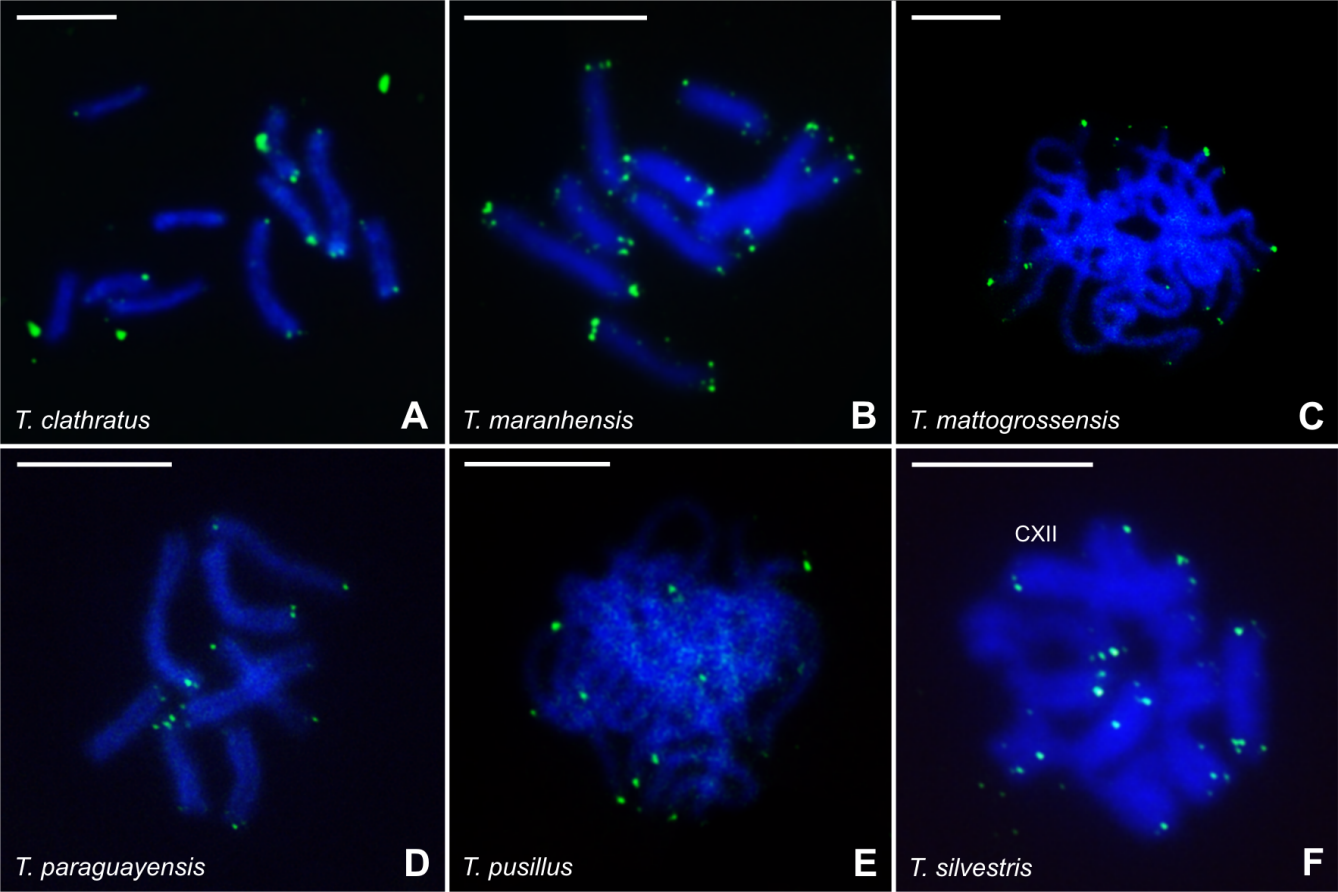
Localization of the (TTAGG)n telomeric region (green signal) in chromosomes of *Tityus* species. (A, B, D, F) Postpachytene cells. (C, E) Pachytene cells. XII = multivalent association of 12 chromosomes. Scale bar = 10μm.

Among the six *Tityus* species, FISH with the (TTAGG)n probe revealed typical telomeric signals in all chromosomes or at least at one chromosome end (Fig 8). Additionally, there was no evidence of positive signals in the interstitial regions of the chromosomes in the analyzed species. Early prophase I nuclei contained brightly labeled regions in one nuclear polo, indicating the clustering of telomeres to form a bouquet. Early prophase I cells exhibited the brightest telomeric signals due to the low degree of chromosome condensation. Postpachytene nuclei also exhibited bright labeling only in the terminal regions of the bivalents and/or chromosomes of the chains (Fig 8A, 8B, 8D and 8F).

## Discussion

In this work, we cytogenetically characterized six out of the eight *Tityus* (*Archaeotityus*) species that occur in Brazil, emphasizing diploid number variability and chromosome behavior during meiosis. These findings represent approximately 20% of the known described species belonging to the subgenus *Archaeotityus*. Representatives of this subgenus are included in the most diverse scorpion genus, *Tityus*, with about 210 described species, all of which are exclusive to the Neotropical region [66]. Although there is no comprehensive phylogenetic hypothesis for this genus, in a recent study of the southernmost American buthids, Ojanguren-Affilastro et al. [67] recovered *Tityus* as a monophyletic clade and also the four studied species of the subgenus *Archaeotityus*. Based on the six species described cytogenetically (*T*. *clathratus*, *T*. *maranhensis*,*T*. *mattogrossensis*, *T*. *paraguayensis*, *T*. *pusillus* and *T*. *silvestris*), *Archaeotityus* scorpions have diploid numbers ranging from 2n = 16 to 2n = 24, with a predominance of 2n = 20 ([29, 68–69], this work). The 2n = 24 reported herein for one population of *T*. *silvestris* is the highest chromosome number for this group.

The extraordinary diploid number diversity recorded for *Tityus* and Buthidae species, in general, is coupled with genome organization. The first sequenced scorpion genome (*Mesobuthus*, Buthidae) suggested the presence of gene duplication events resulting in more than 32,000 protein-coding genes, thus representing more than any sequenced arthropods [70]. Based on genomic and karyotype data, we hypothesize that some chromosomal features may facilitate gene duplication in buthids, i.e., the presence of polyploid cells, holocentric chromosomes, absence of meiotic recombination, and ability to segregate multi-chromosomal associations. Prophase I cells with an increased numberof chromosomes (bivalents and chains) have been observed in *T*. *bahiensis* [50, 71, 72], *T*. *paraguayensis* [29], and *T*. *pusillus* (present work, Fig 5L and 5M). This type of cell appears to recur in buthids and likely arose via endopolyploidy (see [29]). In this manner, some duplicated chromosome or chromosomal segments may have been incorporated into the diploid set, giving rise to changes in diploid number or structural chromosome organization. Holocentricity permits the correct segregation of any chromosomal segment, while achiasmatic chromosomal behavior and the balanced segregation of chromosomal chains avoid the formation of gametes with potentially disadvantageous deletions. These factors may be responsible for chromosome variability, which appears to occur freely in buthids, as well as the expansion of gene families and neofunctionalization, as described by Cao et al. [70] and Sharma et al. [73]. Alternatively, as proposed for angiosperms [74–76], polyploidy/paleopolyploidy followed by repeated disploidy events also lead to the high variability of diploid numbers during meiosis I. In scorpions, however, the whole-genome duplication has been debated (for reviews, see [70, 77]).

Intraspecific variability in diploid number has been recorded in 40% of the 18 karyotyped *Tityus* species [42, 69]. Similar to the species investigated herein, previous studies revealed variations at the inter- and intra-population levels and attributed differences in chromosome number to fission/fusion rearrangements [29, 39, 41, 42, 48]. In specimens of *T*. *clathratus* with 2n = 19, we observed one chromosome that was slightly larger than the others in the diploid set, corroborating the chromosome fusion from 2n = 20. This same rearrangement should occur in specimens of *T*. *mattogrossensis* and *T*. *pusillus* with 2n = 19. However, in these species, the karyotypes with 2n = 19 did not originate only via simple fusion rearrangement because meiotic cells showed multi-chromosomal associations with more than three chromosomes (see Tables 2 and 3). In a simple fusion, the expected result is a trivalent association during meiosis I, as observed in one male specimen of *T*. *mattogrossensis* (UFMT 1369), in *T*. *bahiensis* with 2n = 5, *T*. *curupi* with 2n = 31, and *T*. *obscurus* with 2n = 13 and 2n = 11 [39, 41, 42]. In *T*. *paraguayensis*, it is premature to speculate whether the karyotype with 2n = 16 originated from 2n = 18 by fusion or if 2n = 18 arose from 2n = 16 by fission. Nevertheless, karyotype 2n = 17 is clearly not a hybrid between individuals with 2n = 16 and 2n = 18 because postpachytene cells exhibited a multivalent association with seven rather than three chromosomes. However, the large difference in diploid number between two distant populations (1.040 Km) of *T*. *silvestris*, 2n = 16 in the state of Mato Grosso and 2n = 24 in the state of Amazonas, must be evaluated because these individuals may correspond to distinct taxonomic entities with a low degree of morphological differentiation.

In addition to diploid number variability, all species characterized in this work showed multi-chromosomal associations during meiosis, indicating that at least some individuals are carriers of heterozygous rearrangements. These multivalent associations differed even among specimens with the same chromosome number, such as *T*. *clathratus*, 2n = 19 – 3II+XIII, 4II+XI; *T*. *mattogrossensis*, 2n = 19 – 6II+III+IV, 8II+III; and *T*. *pusillus*, 2n = 19 – 7II+V, 8II+III. Additionally, multivalent associations appeared in individuals lacking odd diploid numbers, such as *T*. *clathratus*, 2n = 20 – 5II+X; *T*. *mattogrossensis*, 2n = 20 – 8II+IV; *T*. *pusillus*, 2n = 20 – 7II+VI; and *T*. *silvestris*, 2n = 16 – 2II+XII. Based on these results, we suggested that independent chromosomal rearrangements (fusion/fission and reciprocal translocations) occurring in different combinations are present in specimens with the same diploid number. This phenomenon is particularly apparent in specimens of *T*. *mattogrossensis* with 8II+IV, which showed significant interpopulational differences regarding the rearranged chromosomes that constituted the quadrivalent association. Furthermore, certain heterozygous rearrangements likely become established in populations, as observed for *T*. *silvestris* from Mato Grosso, in which all examined specimens exhibited 2II+XII. According to Faria and Navarro [17], chromosome rearrangement fixation depends on many factors, such as selection, meiotic drive, and small and semi-isolated demes. In *Tityus* species, all these factors may be present, but given the recurrence of chromosome changes, it is unlikely that rearrangements arose and were fixed only by genetic drift.

The intra-individual variability of the chromosome chains observed in *T*. *clathratus*, *T*. *paraguayensis* and *T*. *pusillus* may have several explanations: 1) a variable degree of heterosynapsis between non-homologous chromosome regions; 2) the early dissociation of the chromosomes constituting the multivalent chains (for details, see 29 and 78); and 3) the persistence of unsynapsed chromosome regions, which are visualized in chains as gaps or open configurations. The two first factors lead to variations in the number of bivalents and/or chromosomes in chains, such as *T*. *clathratus* with 3II+XI, 4II+X, 4II+XI or 4II+X, 4II+XI or 5II+IX, 5II+X, 5II+XI. These events have also been described for other analyzed buthids based on light and electron microscopy observations [29, 39, 78] and appear to be recurrent in other organisms bearing heterozygous rearrangements such as grasshopper, lizard and rodent [79–81]. To confirm whether any other mechanisms of chromosomal change are responsible for intraindividual variation in the number of bivalents without alterations in the number of chromosomes in chains, we determined the DSL for two specimens of *T*. *clathratus* with 3II+X, 4II+X and 5II+X (CHNUFPI 1853) and with 2II+XI, 3II+XI, 4II+XI (CHNUFPI 1863). In both specimens, the comparison of DSL values was not statistically significant, indicating that intra-individual variation in postpachytene cells occurred due to a variety of heterosynaptic interactions between chromosomes.

As mentioned above, the presence of unsynapsed chromosomes segments may also be reflected in the intra-individual variability of chromosome configurations visualized at meiosis I, such as in *T*. *paraguayensis* with 5II+VII and 5II+VIII, and *T*. *pusillus* with 7II+IV and 7II+V. In these two species, when a gap appears on one chromosome, the chain appears to be composed of the largest number of elements. In an elegant study in mice bearing multiple Robertsonian translocations, Manterola et al. [82] demonstrated that unsynapsed chromosomal regions persist until late prophase I, escaping the pachytene checkpoint that ensures correct synapsis. According to the authors, this permissive pachytene checkpoint contributes to the maintenance and spreading of chromosomal translocations intopopulations in an evolutionary context.

To evaluate the effects of multi-chromosomal association on meiotic segregation, we applied the chi-square test in metaphase II cells of individuals with odd diploid numbers. The non-significant occurrence of aneuploid metaphase II nuclei indicated that non-disjunction was negligible in specimens bearing heterozygous rearrangements. As multi-chromosomal chains are widespread among *Tityus* species, we already made assumptions regarding the correct number of chromosomes in metaphase II as a mechanism to avoid unbalanced gametes, which potentially reduce the reproductive fitness of heterozygotes. Although segregation patterns have only been analyzed for certain specimens, these results provide evidence regarding the role of selection in the maintenance of chromosomal rearrangements in scorpions.

Studies examining specific chromosomal regions in scorpions have increased significantly in recent years [29, 39–42, 48, 83–87]. Data regarding the localization of NORs and rDNA has revealed remarkable stability, particularly in *Tityus* species, in which more than 80% of the species presented a similar pattern, i.e., NORs/rDNA in the terminal regions of two chromosomes. Furthermore, in the six species analyzed here, FISH revealed that the 28S rDNA genes were always located in bivalents with a similar size, despite the extensive variability of postpachytene configurations, with the exception of *T*. *silvestris* from Mato Grosso, which exhibited rDNA sites in two chromosomes of the chain. Heckmann et al. [88] discussed the functional interrelationship between holocentricity and terminal NOR position. After revising the positions of NORs in many holocentric species and examining the structures of holocentric chromosomes in *Luzula elegans*, the authors suggested that rDNA sites in distal centromere-free chromosome regions ensure chromosome stability, avoiding breaks. Scorpiones are particularly interesting subjects in which this prediction may be tested because it exhibits both holocentric (Buthidae) and monocentric chromosomes (Bothriuridae, Hormuridae, Scorpionidae and Scorpiopidae).

Detection of the telomeric cluster (TTAGG)n in chromosomes of six *Archaeotityus* species confirmed the presence of this sequence in individual carriers of holocentric chromosomes and multivalent chains. This pentanucleotide sequence has previously been reported for six species: the scorpionid *Heterometrus spinifer* (2n = 49) with monocentric chromosomes [89] and the buthids *Zabius fuscus* (2n = 18), *T*. *confluens* (2n = 5–6), *Tityus curupi* (2n = 32–31), *T*. *obscurus* (2n = 11–16) and *Tityus uruguayensis* (2n = 31) with holocentric chromosomes [40–42, 48]. Thus, the telomeric chromosomal region is conserved despite extensive rearrangements. The synthesis of new telomeres at break points is a feature of holocentric chromosomes, enabling their rapid karyotype evolution through fissions or other rearrangements [90].

## Conclusions

In summary, we demonstrated that karyotype variability in *Tityus (Archaeotityus)* species originated not only via chromosome fission/fusion but also via reciprocal translocation, which gave rise to multi-chromosomal associations during meiosis. Moreover, in the absence of abnormal metaphase II cells, we validated the balanced segregation of chromosomes in individuals bearing heterozygous rearrangements. Finally, based on our analysis of many chromosome characteristics, e.g., holocentricity, achiasmate meiosis, endopolyploidy, ability to segregate heterosynaptic or unsynapsed chromosomes, (TTAGG)n sequence located in terminal regions of rearranged chromosomes, we suggest that the maintenance of multi-chromosomal associations may be evolutionarily advantageous for these species.
